# COVID-19 and Its Psychological Impacts on Healthcare Staff – A Multi-Centric Comparative Cross-Sectional Study

**DOI:** 10.7759/cureus.11753

**Published:** 2020-11-28

**Authors:** Hafsa Shahid, Mobeen Z Haider, Muhammad Taqi, Adnan Gulzar, Zarlakhta Zamani, Tehreem Fatima, Yousra Khalid, Zahoor Ahmed, Hafiza A Nadeem, Faiz Anwer

**Affiliations:** 1 Internal Medicine, King Edward Medical University, Lahore, PAK; 2 Orthopedic Surgery, King Edward Medical University (KEMU)/Mayo Hospital, Lahore, PAK; 3 Internal Medicine, Ameer-ud-Din Medical College, Post-Graduate Medical Institute (PGMI), Lahore, PAK; 4 Internal Medicine, California Institute of Behavioral Neurosciences & Psychology (CIBNP), Fairfield, USA; 5 Internal Medicine, King Edward Medical University/Mayo Hospital, Lahore, PAK; 6 Oncology, Cleveland Clinic, Cleveland, USA

**Keywords:** covid-19, medical staff, psychological impacts, pandemic, quality improvement, mental health, evidence based medicine, primary healthcare workers, infectious disease, internal medicine

## Abstract

Background

Since the first case of coronavirus disease-19 (COVID-19) in Pakistan was reported in February 2020, the medical and paramedical staff has been working on the frontlines to deal with this disease. They have been facing significant strain and stress due to the pandemic, affecting their social, mental, and personal life. The purpose of this study is to investigate the psychological effects of the COVID-19 pandemic, etiology, personal coping mechanisms, and the strategies that are being adopted to reduce stress by the healthcare workers (HCWs) working in COVID-19 dedicated wards (group 2) and compare it with staff working in other departments but not in COVID-19 wards amid this pandemic (group 1) in various hospitals of Lahore, Pakistan.

Methods

The comparative cross-sectional study was designed which included doctors, nurses, and allied health professionals from various hospitals of Lahore, Pakistan. A questionnaire was designed which consisted of five sections, and 51 questions. A Chi-square test was used to compare the responses between these two groups.

Results

The study questionnaire was submitted by 200 participants, 100 responses for each group (see the Appendix). In group 1, HCWs not working in COVID-19 dedicated floors were afraid of getting infected, transmitting the infection to their families and concerned about using personal protective equipment (PPE) improperly. They reported a lack of concentration and tense muscles. The coping mechanisms of this group were exercise, strict precautions at work, and social distancing measures. While HCWs serving in COVID-19 dedicated wards were concerned and afraid of putting their families at risk by working in the high-risk environment; the major stresses in this group were: lack of knowledge about proper strategies for treatment, they faced insecurity due to physical and verbal violence by caretakers of COVID-19 patients, and lack of concentration. The coping mechanism was the support of their families and taking strict precautions, with self-isolation if required, to avoid any disease transmission to their families. The proposed strategies to be implemented included teaching skills for self-rescue as well as the implementation of policies at the administrative level to reduce working hours and frequent shift rotation.

Conclusion

The COVID-19 outbreak posed a great deal of mental stress among HCWs working on the COVID-19 floor as well as those serving in other departments of the hospital. The HCWs from group 1 were most afraid of getting infected and putting family members at risk, experienced tense muscles and lack of concentration, coped their stress by exercise and being more vigilant, and suggested the strategies of teaching skills for self-rescue and better community awareness. While the staff from the second group were most afraid of being the source of infection and violence from the caretakers of patients, experienced tense muscles, used family support, and strict isolation measures as coping mechanisms and suggested the strategies of self-rescue and increase in wages of directly exposed healthcare workers to deal with such pandemics in future in a better way.

## Introduction

In November 2019, a pneumonia outbreak of unknown cause occurred in Wuhan, China. Seafood was reported to be the primary source of the disease [1]. On January 30, 2020, the COVID-19 pandemic was labeled as a public health emergency of international concern by the World Health Organization (WHO) [2]. In Pakistan, the cases started to be reported in February 2020. According to Dawn News, a total of 231,017 cases were reported till July 5, 2020. This pandemic created a lot of panic in the general public and induced stress in all healthcare workers - whether working in COVID-19 wards or any other department of the hospitals. Medical practitioners, being the front liners in every pandemic, have been facing psychological issues. As estimated from the previous pandemic of the Ebola virus in 2014, healthcare workers faced major psychiatric problems involving obsessive-compulsive disorder (OCD), paranoia, and hostility [3]. Healthcare staff is afraid of getting infected and transmitting the virus to their families. Lack of appreciation by administrative authorities and a rise in violence against medical staff further precipitate the mental distress among healthcare workers. The sudden increase in duty hours and shift rotation results in physician burnout. The behavior of their seniors and lack of support from the family leads to further nervous exhaustion. Hence, COVID-19 has a major psychological impact on the medical and paramedical staff. Although there are many studies regarding the psychological effects of COVID-19 on the medical staff, its psychological effects in the population of medical and paramedical staff of Lahore are still unknown. Also, the interventions adopted by the healthcare workers and their impact on relieving their stress are unclear till yet. Furthermore, the steps that must be taken by the administrative authorities to mitigate such psychological conditions have also not been established yet. In our study, we will compare the prevailing psychological symptoms in HCWs; measures are taken to relieve such manifestations and the suggestions regarding actions should be considered by higher authorities for medical and paramedical staff working in COVID-19 wards and those healthcare workers who don’t attend COVID-19 wards but are working in other departments of hospitals during this pandemic.

## Materials and methods

A comparative cross-sectional study was conducted including doctors, nurses, and allied health professionals working in Lahore, Pakistan to compare the psychological impacts and their causes, personal coping mechanisms, and strategies that are being adopted to minimize the effect of a pandemic on mental health amid the time of current COVID-19 pandemic.

Study participants

The sample size was calculated by an online sample size calculator by using the confidence interval of 95% and the margin of error as 5%. It was assumed that 10% of all the working medical staff had worked in COVID-19 wards while 90% of the staff was still working in other departments of the hospitals. The ratio for each group was 1:1. So, we selected the sample size of 100 for group 1 (the staff working in other departments of the hospital and have not worked in COVID-19 wards yet) and the sample size of 100 for the second group (the staff working or have worked in COVID-19 wards), making total sample size to be 200 participants.

Two groups were formulated to compare these variables:

Group 1: All the medical and paramedical staff who have not worked in COVID-19 wards yet, but they are working in other departments of various hospitals of Lahore during this time of Pandemic.

Group 2: All the medical and paramedical staff who have worked or been working in COVID-19 wards in various hospitals of Lahore.

Sample selection

Inclusion Criteria

Group 1: All those healthcare workers who had worked/have been working in other departments of hospitals and had never worked in COVID-19 wards during this pandemic.

Group 2: All those healthcare workers who worked in COVID-19 wards.

Exclusion criteria

Group 1: All those healthcare workers who worked in COVID-19 wards as well as other departments of the hospital. Those healthcare workers who had worked in COVID-19 wards.

Group 2: All those healthcare workers who did not work in COVID-19 wards.

The questionnaire included 51 questions and all the participants were to comprehend the questions and then answer them on their own.

Statistical analysis

The collected data were analyzed statistically by using IBM SPSS Statistics version 23 (IBM Corp., Armonk, NY). The Chi-Square χ2 test was performed to compare the responses between the two afore-mentioned groups based on their gender and age-groups for all the sections of the responses, except demographics. Descriptive statistics were applied to present the data collected in the form of mean, median, and standard deviation. A p-value of <0.05 was considered significant.

## Results

Characteristics of the population under study

Data were collected from a total of 200 healthcare workers; 100 for each group according to the sample size calculated. Out of these 200, 111 (55.5%) were males and 89 (44.5%) were females. In group 1, 46 (46%) were males and 54 (54%) were females. In group 2, 65 (65%) were males and 35 (35%) were females. According to the profession, 90% of the sample population consisted of doctors. In the first group, 88/100 (88%) consisted of doctors; in group 2, 92/100 (92%) were doctors. Most of the participants were of the age group 23-35 years. In group 1, 88/100 (88%) fall under the given age group; while in the second group, 92/100 (92%) belong to this age group. Regarding the workplace, 65% of the healthcare professionals were from Mayo Hospital, Lahore. Most of them belonged to the internal medicine department (39.5%). In group 1, 19/100 (19%) were from the medicine department and in group 2, 60/100 (60%) were from the medicine department. The demographic characteristics have been mentioned in the table given below (Table 1).

**Table 1:** Characteristics of the population under study For group 1: n = 100 For group 2: n = 100

	GROUP 1	GROUP 2
Gender, n (%)		
Male	46 (46%)	65 (65%)
Female	54 (54%)	35 (35%)
Profession, n (%)		
Doctors	88 (88%)	92 (92%)
Nurses	2 (2%)	5 (5%)
Allied Health Professionals	10 (10%)	3 (3%)
Age-groups (years), n (%)		
Standard Deviation (SD)	1.01384	0.72829
20-25	55 (55%)	20 (20%)
26-30	32 (32%)	56 (56%)
31-35	8 (8%)	21 (21%)
36-40	2 (2%)	3 (3%)
41-45	1 (1%)	0 (0%)
46-50	2 (2%)	0 (0%)
Workplace, n (%)		
Mayo Hospital, Lahore	60 (60%)	70 (70%)
Sir Ganga Ram Hospital, Lahore	8 (8%)	5 (5%)
Sheikh Zaid Hospital, Lahore	7 (7%)	3 (3%)
Shalamar Hospital, Lahore	3 (3%)	2 (2%)
Services Hospital, Lahore	6 (6%)	4 (4%)
Jinnah Hospital, Lahore	3 (3%)	3 (3%)
General Hospital, Lahore	3 (3%)	3 (3%)
Other Hospitals of Lahore	10 (10%)	10 (10%)
Department, n (%)		
Medicine	19 (19%)	60 (60%)
Surgery	19 (19%)	2 (2%)
ENT	6 (6%)	2 (2%)
Neurology	4 (4%)	3 (3%)
Anesthesia	3 (3%)	3 (3%)
Other departments of Hospitals	49 (49%)	30 (30%)

The fears faced by the medical and paramedical staff

The fears faced by the medical staff during this pandemic are mentioned in Table 2. These fears in both the groups, further classified under “age group” and “gender”, are compared separately for each subgroup by applying the Chi-Square test; results were significant in both groups.

**Table 2:** Fears faced by healthcare workers All values are in percentage except p-values. Answer choices (SA = Strongly agree, A = Agree, N = Neutral, DA = Disagree, SDA = Strongly Disagree), Gender (M = male, F = Female).

Questions		Group 1										Group 2									
	Answer options	Age Group							Gender			Age Group							Gender		
Fear of:		20-25	26-30	31-35	36-40	41-45	46-50	p-value	Male	Female	p-value	20-25	26-30	31-35	36-40	41-45	46-50	p-value	Male	Female	p-value
1- Getting infected	SA	60	31.1	6.7	0	0	2.2	0.026*	46.7	53.3	0.273	24	54	18	4			0.033*	72	28	0.287
	A	46.5	37.2	11.6	0	0	2.3		51.2	48.8		16.7	55.6	27.8	0				63.9	36	
	N	66.7	16.7	0	16.7	0	0		25	75		9.1	72.7	18.2	0				45.5	54.5	
	DA	0	0	0	0	0	0		0	0		0	100	0	0				0	100	
	SDA	0	0	0	0	0	0		0	0		50	0	0	50				50	50	
2- Being quarantined	SA	55.1	31	10.3	0	0	3.4	0.950*	58.6	41.4	0.205	21.4	60.7	14.3	3.6			0.029*	64.3	35.7	0.893
	A	53.3	33.3	8.9	2.2	0	2.2		37.8	62.2		25.6	51.3	20.5	2.6				69.2	30.8	
	N	58.8	23.5	5.9	5.9	5.9	0		35.3	64.7		16.6	62.5	20.8	0				58.3	41.7	
	DA	50	50	0	0	0	0		62.5	37.5		0	57.1	42.8	0				71.4	28.6	
	SDA	100	0	0	0	0	0		100	0		0	0	50	50				50	50	
3- Being the source of infection	SA	61.2	29.8	7.5	0	0	1.5	<0.001*	44.7	55.2	0.66	21.1	53.5	23.9	1.4			0.685	70.4	29.6	0.035*
	A	41.7	37.5	12.5	4.2	0	4.2		45.8	54.2		20	56	16	8				60	40	
	N	42.8	42.8	0	14.9	0	0		57.1	42.8		0	100	0	0				0	100	
	D	100	0	0	0	0	0		0	100		0	100	0	0				0	100	
	SDA	0	0	0	0	100	0		100	0		0	0	0	0				0	0	
4- Putting family at risk	SA	53.7	36.2	8.7	0	0	1.2	0.003*	47.5	52.5	0.503	23.7	53.7	20	2.5			0.743	70	30	0.09
	A	58.8	17.6	5.9	5.8	5.9	5.9		35.3	64.7		5.9	64.7	23.5	5.9				41.2	58.8	
	N	66.7	0	0	33.3	0	0		66.7	33.3		0	0	100	0				100	0	
	DA	0	0	0	0	0	0		0	0		0	100	0	0				100	0	
	SDA	0	0	0	0	0	0		0	0		0	100	0	0				0	100	
5- Using PPE improperly	SA	60	32	8	0	0	0	<0.001*	44	56	0.146	21.7	65.2	13	0			0.132	65.2	34.8	0.148
	A	57.8	28.9	13.3	0	0	0		53.3	46.7		23.8	57.1	16.7	2.4				69	30.9	
	N	52.9	29.4	0	11.7	0	5.9		23.5	76.5		19	52.4	23.8	4.8				71.4	28.6	
	DA	45.5	45.4	0	0	9.1	0		45.4	54.5		9	45.4	45.4	0				54.5	45.4	
	SDA	0	50	0	0	0	50		100	0		0	33.3	33.3	33.3				0	100	
6- Losing a patient	SA	63.9	19.4	13.9	0	0	2.8	<0.001*	44.4	55.6	0.722	31.7	58.5	7.3	2.4			0.118	56	43.9	0.194
	A	55.3	38.3	4.2	0	2.1	0		46.8	53.2		31.7	58.5	7.3	2.4				72	28	
	N	30	40	10	20	0	0		60	40		16.6	50	33.3	0				50	50	
	DA	50	50	0	0	0	0		33.3	66.7		0	33.3	66.7	0				100	0	
	SDA	0	0	0	0	0	100		0	100		0	0	0	0				0	0	
7- Lock-down issues	SA	66.7	21.2	6.1	0	3	3	0.64	39.4	60.6	0.052	21.4	57.1	17.8	3.6			0.773	71.4	28.6	0.388
	A	38.2	38.2	14.7	5.8	0	2.9		64.7	35.3		20.7	55.2	17.2	6.9				55.2	44.8	
	N	64.7	29.4	5.9	0	0	0		29.4	70.6		16	60	24	0				72	28	
	DA	60	40	0	0	0	0		33.3	66.7		17.6	52.9	29.4	0				64.7	35.3	
	SDA	0	100	0	0	0	0		100	0		100	0	0	0				0	100	
8- Lack of knowledge about Rx	SA	75	16.7	8.3	0	0	0	0.025*	37.5	62.5	0.277	40	46.7	6.7	6.7			0.006*	46.7	53.3	0.114
	A	58.1	34.9	4.6	0	0	2.3		41.9	58.1		21.4	71.4	7.1	0				57	42.8	
	N	26.7	40	13.3	13.3	6.7	0		46.7	53.3		17.2	44.8	34.9	3.4				79.3	20.7	
	DA	41.7	41.7	16.7	0	0	0		58.3	41.7		8	60	32	0				72	28	
	SDA	55	32	8	2	1	2		83.3	16.7		33.3	33.3	0	33.3				33.3	66.7	
9- Exhaustion d/t increase working hours	SA	64.3	28.6	7.1	0	0	0	0.126	42.8	57.1	0.913	17.2	65.5	13.8	3.4			0.228	65.5	34.5	0.338
	A	45.4	36.4	18.2	0	0	0		50	50		21.9	60.9	12.2	4.9				73.2	26.8	
	N	51.7	34.5	3.4	6.9	3.4	0		44.8	55.2		29.4	35.3	35.3	0				58.8	41.2	
	DA	73.3	20	0	0	0	6.7		53.3	46.7		8.3	50	41.7	0				50	50	
	SDA	16.7	50	16.7	0	0	16.7		33.3	66.7		0	0	100	0				0	100	
10- Violence by care-takers of patients	SA	60	28	10	0	0	2	0.171	56	44	0.198	25	59.6	13.5	1.9			0..023*	63.5	36.5	0.806
	A	50	35.3	8.8	0	2.9	2.9		32.3	67.6		15	60.6	21.2	3				63.6	36.4	
	N	58.3	33.3	0	8.3	0	0		41.7	58.3		22.2	44.4	22.2	11.1				66.7	33.3	
	DA	33.3	33.3	0	33.3	0	0		33.3	66.7		0	16.7	83.3	0				83.3	16.7	
	SDA	0	100	0	0	0	0		100	0		0	0	0	0				0	0	

In group 1, HCWs were afraid of getting infected (p = 0.026), being the source of infection for their fellows and family (p < 0.001), putting their family members at risk (p = 0.003), using PPE improperly as they had not been guided about its usage (p < 0.001), losing the patients as medical attention had been drawn to the pandemic (p < 0.001) and the lack of knowledge about the treatment of this disease (p = 0.025).

In group 2, the healthcare workers were scared of being the source of infection (p = 0.035), getting themselves infected while working in the COVID-19 wards (p = 0.033), being isolated (p = 0.029), lack of knowledge about the treatment of this disease (p = 0.006) and the violence by the caretakers of the patients infected with COVID-19 (p = 0.023).

Psychological symptoms experienced by the healthcare workers

The psychological symptoms that have been investigated in this study are listed in Table 3. These symptoms were compared in both groups and further subclassified under age-groups and gender. There were a total of eight symptoms that were investigated in the questionnaire. A total of three results were found significant after applying the Chi-Squared test.

**Table 3:** Symptoms experienced by healthcare workers All Values are in percentage except p-values. Answer choices (SA = Strongly agree, A = Agree, N = Neutral, DA = Disagree, SDA = Strongly Disagree), Gender (M = Male, F = Female).

		Group 1										Group 2									
Questions		Age group							Gender			Age group							Gender		
	Answer choices	20-25	26-30	31-35	36-40	41-45	46-50	p-value	Male	Female	p-value	20-25	26-30	31-35	36-40	41-45	46-50	p-value	Male	Female	p-value
1- Insomnia	SA	64.3	28.6	7.1	0	0	0	0.801	50	50	0.92	18.7	43.7	25	12.5			0.291	62.5	37.5	0.783
	A	50	41.7	4	0	0	4.2		41.7	58.3		27.6	65.5	6.9	0				62.1	37.9	
	N	56	24	12	4	4	0		52	48		18.2	50	27.3	4.5				63.6	36.4	
	DA	55	31	10.3	0	0	3.4		41.4	58.6		18.5	55.5	25.9	0				74.1	25.9	
	SDA	50	37.5	0	12.5	0	0		50	50		0	66.6	33.3	0				50	50	
2- Headache	SA	71.4	0	14.3	0	0	14.3	0.092	42.8	57.1	0.844	18.7	62.5	12.5	6.25			0.172	50	50	0.533
	A	54.7	38	7.1	0	0	0		45.2	54.7		24.2	54.5	21.2	0				60.6	39.4	
	N	48	36	16	0	0	0		48	52		27.7	38.9	22.2	11.1				72.2	27.8	
	DA	60	25	0	5	5	5		40	60		15.4	69.2	15.4	0				73.1	26.9	
	SDA	50	33.3	0	16.7	0	0		66.7	33.3		0	42.8	57.1	0				71.4	28.6	
3- Lack of concentration	SA	75	16.7	8.3	0	0	0	0.003*	58.3	41.7	0.164	36.4	63.6	0	0			0.051	45.4	54.5	0.637
	A	44.2	44.2	9.3	2.3	0	0		41.8	58.1		16.2	64.8	13.5	5.4				67.5	32.4	
	N	52.9	35.3	11.7	0	0	0		52.9	47		12	56	28	4				72	28	
	DA	72.7	18.2	4.5	0	0	4.5		31.8	68.2		36.8	42.1	21	0				63.1	36.8	
	SDA	33.3	16.7	0	16.7	16.7	16.7		83.3	16.7		0	37.5	62.5	0				62.5	37.5	
4- Restlessness	SA	78.6	7.1	14.3	0	0	0	0.361	50	50	0.639	20	60	20	0			0.279	73.3	26.7	0.613
	A	44.7	42.1	7.9	2.6	0	2.6		47.4	52.6		27.9	53.5	13.9	4.6				60.5	39.5	
	N	57	28.6	9.5	0	4.7	0		33.3	66.7		11.1	72.2	11.1	5.5				55.5	44.4	
	DA	52.4	38	4.7	0	0	4.7		47.6	52.4		15.8	47.4	36.8	0				73.7	26.3	
	SDA	66.7	16.7	0	16.7	0	0		66.7	33.3		0	40	60	0				80	20	
5- Fatigue	SA	68.2	18.2	13.6	0	0	0	0.617	54.5	45.4	0.304	21.4	64.3	14.3	0			0.914	57.1	42.8	0.53
	A	52.3	34	6.8	2.3	2.3	2.3		34	65.9		22.9	52	20.8	4.2				64.6	35.4	
	N	46	38.5	15.4	0	0	0		53.8	46		15.4	53.8	23.1	7.7				84.6	15.4	
	DA	53.3	40	0	0	0	6.7		53.3	46.7		11.1	55.5	33.3	0				66.7	33.3	
	SDA	50	33.3	0	16.7	0	0		66.7	33.3		0	50	50	0				50	50	
6- Worried	SA	61	16.7	16.7	0	0	5.5	0.967	55.5	44.4	0.929	33.3	46.7	16.7	3.3			0.653	63.3	36.7	0.597
	A	54.8	32.2	8	1.6	1.6	1.6		43.5	56.4		18.6	58.1	20.9	2.3				62.8	37.2	
	N	50	43.7	0	6.2	0	0		43.7	56.2		14.3	64.3	21.4	0				71.4	28.6	
	DA	50	50	0	0	0	0		50	50		0	58.3	33.3	8.3				75	25	
	SDA	50	50	0	0	0	0		50	50		0	100	0	0				0	100	
7- Tense muscles	SA	61	16.7	16.7	0	0	5.5	0.023*	57	42.8	0.017*	33.3	46.7	16.7	3.3			0.306	53.8	46.1	0.355
	A	54.8	32.2	8	1.6	1.6	1.6		36.4	63.6		18.6	58.1	20.9	2.3				59.4	40.6	
	N	50	43.7	0	6.2	0	0		26.9	73		14.3	64.3	21.4	0				72	28	
	DA	50	50	0	0	0	0		65.4	34.6		0	58.3	33.3	8.3				76	24	
	SDA	50	50	0	0	0	0		75	25		0	100	0	0				40	60	
8- Panic attacks	SA	75	0	25	0	0	0	0.88	50	50	0.336	28.6	57.1	0	14.3			0.431	42.8	57.1	0.458
	A	52.9	29.4	5.8	5.9	0	5.9		35.3	64.7		22.2	66.7	11.1	0				66.7	33.3	
	N	48	40	12	0	0	0		52	48		21.4	53.6	25	0				57.1	42.8	
	DA	59.4	29.7	5.4	0	2.7	2.7		37.8	62.2		20	54.3	20	5.7				74.3	25.7	
	SDA	52.9	35.2	5.9	5.9	0	0		64.7	35.3		8.3	50	41.7	0				66.7	33.3	

In group 1, healthcare workers were found to be experiencing tense muscles (p = 0.017) and a lack of concentration during the pandemic (p = 0.03).

In group 2, the lack of concentration was the prevailing symptom (p = 0.051) followed by headache (p = 0.172).

Personal coping mechanisms

The personal coping mechanisms adopted by the medical and paramedical staff to get relief from such psychological effects of the pandemic that were investigated are mentioned in Table 4. These coping mechanisms were compared in both the group, further classified under age-groups and gender. There were a total of 12 coping mechanisms that were stated in the questionnaire. After applying the Chi-Square test, a total of five results were found significant.

**Table 4:** Personal coping mechanism of healthcare workers ARD = Anxiety Relieving Drugs. All Values are in percentage except p-values. Answer choices (SA = Strongly agree, A = Agree, N = Neutral, DA = Disagree, SDA = Strongly disagree).

		Group 1										Group 2									
Question		Age group							Gender			Age group							Gender		
	Answer choices	20-25	26-30	31-35	36-40	41-45	46-50	p-value	M	F	p-value	20-25	26-30	31-35	36-40	41-45	46-50	p-value	M	F	p-value
1- Used ARD	No	54	33	8	2	1	2	0.972	45	55	0.329	18	57	22	2			0.485	68	32	0.127
	Yes	63	25	13	0	0	0		63	38		31	46	15	8				46	54	
2- Did Exercise	No	63	32	0	2	2	0	0.096	32	68	0.017*	21	47	26	7			0.113	67	33	0.657
	Yes	49	32	14	2	0	3		56	44		19	63	18	0				63	37	
3- Appreciation by fellows	SA	53	27	13	0	0	7	0.768	53	47	0.752	23	65	12	0			0.36	62	38	0.579
	A	57	31	8	0	2	2		41	59		24	49	22	5				59	41	
	N	48	37	7	7	0	0		52	48		5	57	33	5				76	24	
	DA	71	29	0	0	0	0		43	57		27	64	9	0				73	27	
	SDA	0	0	0	0	0	0		0	0		0	0	100	0				100	0	
4- Family support	SA	64	23	10	0	0	3	0.502	38	62	0.443	22	56	20	2			0.050*	66	34	0.289
	A	48	41	6	2	2	2		48	52		19	57	24	0				70	30	
	N	60	0	20	20	0	0		60	40		13	63	13	13				50	50	
	DA	100	0	0	0	0	0		100	0		33	33	33	0				67	33	
	SDA	0	100	0	0	0	0		100	0		0	50	0	50				0	100	
5- Motivation by patients	SA	79	14	7	0	0	0	0.551	43	57	0.888	29	59	12	0			0.577	65	35	0.964
	A	40	46	11	0	0	3		49	51		16	57	25	2				64	36	
	N	59	25	6	6	3	0		41	59		22	57	13	9				65	35	
	DA	63	25	6	0	0	6		50	50		20	53	27	0				67	33	
	SDA	33	67	0	0	0	0		67	33		0	0	100	0				100	0	
6- Positive attitude by seniors	SA	64	23	5	0	5	5	0.86	45	55	0.445	15	62	15	8			0.369	65	35	0.677
	A	62	28	5	3	0	3		38	62		19	55	24	2				67	33	
	N	38	42	17	4	0	0		50	50		11	63	26	0				53	47	
	DA	54	38	8	0	0	0		54	46		40	50	10	0				80	20	
	SDA	50	50	0	0	0	0		100	0		67	0	33	0				67	33	
7- Being more vigilant	SA	69	8	15	0	0	8	0.035*	46	54	0.992	27	54	15	4			0.939	69	31	0.907
	A	53	34	10	2	0	0		45	55		16	60	22	2				62	38	
	N	43	48	0	4	4	0		48	52		19	50	25	6				69	31	
	DA	83	0	0	0	0	17		50	50		33	33	33	0				67	33	
	SDA	0	0	0	0	0	0		0	0		0	0	0	0				0	0	
8- Strict isolation measures	SA	68	21	5	0	0	5	0.025*	37	63	0.749	25	58	13	4			0.353	63	38	0.758
	A	55	31	14	0	0	0		48	52		14	68	16	2				68	32	
	N	45	45	0	7	3	0		48	52		22	39	35	4				57	43	
	DA	67	22	0	0	0	11		44	56		25	38	38	0				75	25	
	SDA	0	0	100	0	0	0		100	0		100	0	0	0				100	0	
9- Self isolation measures	SA	59	29	6	0	0	6	0.708	53	47	0.685	16	68	12	4			0.037*	68	32	0.058
	A	54	31	11	2	2	0		43	57		24	61	16	0				69	31	
	N	63	32	0	5	0	0		42	58		13	38	38	13				38	63	
	DA	40	40	10	0	0	10		60	40		25	25	50	0				88	13	
	SDA	0	0	0	0	0	0		0	0		0	0	0	0				0	0	
10- Positive self attitude	SA	62	27	8	0	0	4	0.983	42	58	0.922	20	67	10	3			0.561	70	30	0.676
	A	49	37	8	3	2	2		46	54		16	56	25	2				65	35	
	N	67	22	11	0	0	0		56	44		33	33	25	8				50	50	
	DA	100	0	0	0	0	0		50	50		33	33	33	0				67	33	
	SDA	0	0	0	0	0	0		0	0		0	0	0	0				0	0	
11- Using immunity boosters	SA	63	21	8	4	0	4	0.545	42	58	0.065	19	67	11	4			0.715	52	48	0.065
	A	51	36	10	3	0	0		33	67		24	52	21	2				67	33	
	N	67	21	8	0	4	0		54	46		11	50	33	6				67	33	
	DA	27	64	0	0	0	9		73	27		30	40	30	0				100	0	
	SDA	50	50	0	0	0	0		100	0		0	100	0	0				33	67	
12- Religious convictions	SA	57	26	13	4	0	0	0.933	39	61	0.251	19	54	23	4			0.991	54	46	0.487
	A	45	40	8	3	3	3		48	53		20	61	17	2				66	34	
	N	70	22	4	0	0	4		43	57		21	54	21	4				71	29	
	DA	60	40	0	0	0	0		40	60		20	40	40	0				80	20	
	SDA	50	25	25	0	0	0		100	0		0	0	0	0				0	0	

In group 1, healthcare workers directed towards exercise (p = 0.017), being more vigilant (p = 0.035), and strict isolation measures (p = 0.025) to get rid of these psychological symptoms.

In group 2, family support was the major factor to relieve their symptoms (p = 0.05). Using strict self-isolation measures to decrease the risk of spreading infection also helped to relieve the stress of transmitting the infection to their families (p = 0.037).

Strategies that should be implemented

The strategies that should be implemented by higher authorities in future pandemics to reduce its psychological effects on healthcare workers are mentioned in Table 5. These strategies were compared in both groups, further classified under age-groups and gender. There were a total of 14 strategies that were stated in the questionnaire. A Chi-Square test was applied to each strategy.

**Table 5:** Strategies to reduce mental stress All values are in percentage except p-values. Answer choices (SA = Strongly agree, A = Agree, N = Neutral, DA = Disagree, SDA = Strongly disagree), Gender (M = male, F = Female).

		Group 1										Group 2									
Question		Age group							Gender			Age group							Gender		
	Answer Choices	20-25	26-30	31-35	36-40	41-45	46-50	p-value	M	F	p-value	20-25	26-30	31-35	36-40	41-45	46-50	p-value	M	F	p-value
1- Community awareness	SA	56	24	13	2	2	3	0.788	50	50	0.242	17	61	19	3			0.69	69	31	0.329
	A	53	43	0	3	0	0		37	63		25	54	17	4				50	50	
	Neutral	50	50	0	0	0	0		33	67		20	40	40	0				60	40	
	DA	50	50	0	0	0	0		100	0		50	25	25	0				75	25	
	SDA	0	0	0	0	0	0		0	0		0	33	67	0				100	0	
2- Self rescue skills	SA	62	26	9	2	0	2	0.93	34	66	0.116	23	58	16	4			0.126	63	37	0.099
	A	43	41	9	2	2	2		59	41		15	61	24	0				58	42	
	N	75	25	0	0	0	0		50	50		0	50	33	17				100	0	
	DA	100	0	0	0	0	0		33	67		50	0	50	0				100	0	
	SDA	0	0	0	0	0	0		0	0		0	0	0	0				0	0	
3- Strict infection control guidelines	SA	63	20	10	2	2	2	0.976	49	51	0.413	19	58	21	2			0.779	65	35	0.355
	A	44	44	8	3	0	3		41	59		20	57	20	3				57	43	
	N	57	43	0	0	0	0		71	29		14	29	43	14				57	43	
	DA	50	50	0	0	0	0		25	75		30	60	10	0				90	10	
	SDA	100	0	0	0	0	0		0	100		0	100	0	0				100	0	
4- Media sources	SA	58	29	7	2	2	2	0.999	51	49	0.392	20	61	17	2			0.181	67	33	0.111
	A	51	35	8	3	0	3		49	51		23	60	17	0				60	40	
	N	47	40	13	0	0	0		33	67		10	40	30	20				40	60	
	DA	100	0	0	0	0	0		0	100		13	50	38	0				100	0	
	SDA	100	0	0	0	0	0		0	100		33	33	33	0				67	33	
5- Psychotherapist consultation	SA	64	21	11	0	0	4	0.236	50	50	0.679	24	48	24	5			0.916	57	43	0.691
	A	33	48	9	6	3	0		52	48		17	60	20	3				60	40	
	N	61	32	7	0	0	0		43	57		21	58	17	4				75	25	
	DA	75	13	0	0	0	13		25	75		24	59	18	0				71	29	
	SDA	100	0	0	0	0	0		33	67		0	33	67	0				67	33	
6- Support for frontline HCWs	SA	54	30	11	3	1	1	0.916	49	51	0.221	21	51	24	3			0.755	64	36	0.965
	A	59	37	0	0	0	4		33	67		10	71	14	5				67	33	
	N	0	100	0	0	0	0		100	0		40	60	0	0				60	40	
	DA	0	0	0	0	0	0		0	0		0	0	0	0				0	0	
	SDA	100	0	0	0	0	0		0	0		25	50	25	0				75	25	
7- Optimal increase in wages	SA	52	31	10	4	2	2	0.998	52	48	0.295	22	55	21	1			0.123	66	34	0.13
	A	56	34	6	0	0	3		41	59		11	56	22	11				56	44	
	N	67	25	8	0	0	0		25	75		0	100	0	0				25	75	
	DA	0	100	0	0	0	0		100	0		60	40	0	0				100	0	
	SDA	67	33	0	0	0	0		67	33		0	50	50	0				83	17	
8- Reduce working hours	SA	47	34	11	4	0	4	0.957	49	51	0.811	19	60	19	2			0.805	67	33	0.436
	A	60	33	3	0	3	0		40	60		17	53	25	6				61	39	
	N	71	21	7	0	0	0		50	50		100	0	0	0				0	100	
	DA	50	50	0	0	0	0		50	50		33	67	0	0				67	33	
	SDA	100	0	0	0	0	0		0	100		33	33	33	0				100	0	
9- Shift rotation of HCWs	SA	48	28	14	4	2	4	0.687	52	48	0.337	23	55	20	2			0.408	63	37	0.884
	A	54	44	3	0	0	0		38	62		9	63	22	6				66	34	
	N	88	13	0	0	0	0		50	50		33	50	17	0				67	33	
	DA	100	0	0	0	0	0		100	0		100	0	0	0				100	0	
	SDA	100	0	0	0	0	0		0	100		0	0	100	0				100	0	
10- Communication with directives	SA	53	24	14	4	2	4	0.807	53	47	0.405	20	57	22	2			0.942	67	33	0.214
	A	55	42	3	0	0	0		42	58		16	58	21	5				58	42	
	N	50	50	0	0	0	0		38	63		33	50	17	0				83	17	
	DA	100	0	0	0	0	0		0	100		50	25	25	0				100	0	
	SDA	100	0	0	0	0	0		0	100		0	100	0	0				0	100	
11- Adequate training	SA	52	29	12	3	2	2	0.813	55	45	0.1	21	57	19	3			0.698	61	39	0.785
	A	62	32	3	0	0	3		32	68		21	63	16	0				74	26	
	N	33	67	0	0	0	0		50	50		14	43	29	14				71	29	
	DA	100	0	0	0	0	0		0	100		17	50	33	0				67	33	
	SDA	0	0	0	0	0	0		0	0		0	0	100	0				100	0	
12- Measures to prevent HCW infection	SA	57	25	11	3	2	2	0.904	48	52	0.203	21	53	24	3			0.846	66	34	0.677
	A	52	41	3	0	0	3		34	66		16	68	11	5				58	42	
	N	43	57	0	0	0	0		71	29		0	67	33	0				67	33	
	DA	0	0	0	0	0	0		0	0		50	50	0	0				100	0	
	SDA	100	0	0	0	0	0		100	0		0	0	0	0				0	0	
13- Accurate information to public	SA	58	24	11	3	1	3	0.336	49	51	0.222	21	51	25	3			0.851	67	33	0.41
	A	45	55	0	0	0	0		32	68		18	68	9	5				59	41	
	N	50	50	0	0	0	0		67	33		0	100	0	0				0	100	
	DA	0	0	0	0	0	0		0	0		0	100	0	0				100	0	
	SDA	0	0	0	0	0	0		0	0		0	0	0	0				0	0	
14- Measures to bust myths	SA	55	28	11	3	1	1	0.514	49	51	0.351	22	53	23	3			0.817	66	34	0.673
	A	58	38	0	0	0	4		42	58		11	67	17	6				56	44	
	N	0	100	0	0	0	0		0	100		0	100	0	0				100	0	
	DA	0	0	0	0	0	0					100	0	0	0				100	0	
	SDA	0	0	0	0	0	0					0	100	0	0				100	0	

According to group 1, skills for self-rescue (p = 0.116), better community awareness (p = 0.242), and adequate training on infection control (p = 0.1), and strong measures to bust myths (p = 0.351) must be implemented.

As suggested by group 2, skills for self-rescue (p = 0.099), optimal increase in wages of HCWs working in high-risk units (p = 0.123), and dissemination of accurate information by media sources (p = 0.41) must be applied.

## Discussion

In comparison with the general population, health care workers are facing tremendous amounts of stress as they are involved in the direct management of patients with COVID-19. Due to the increased risk of exposure, frontline health care workers may experience symptoms of mental health problems such as anxiety, insomnia, physical and mental exhaustion, and other forms of psychological distress [4]. We performed a questionnaire survey to investigate and compare the fears experienced by the health care workers, their association with the adverse physical and psychological outcomes, and the coping mechanisms adopted by HCWs. COVID-19 pandemic posed major psychological stress for the medical and paramedical staff working in various hospitals of Lahore, Pakistan. The teams of healthcare workers not directly working in COVID-19 wards feared a lot about getting infected, being the source of infection for others, increasing the risk of infection for families, using PPE improperly, poor patient outcomes due to the lack of knowledge about the treatment. They experienced the symptoms of tense muscles and lack of concentration. The personal coping mechanisms included exercise, and being more vigilant with preventive measures. The strategies advised by them were better community awareness and proper staff training on infection control. Those healthcare workers who were directly providing care to COVID-19 patients were facing the fears of getting infected, spreading the infection to other patients, and their families. They were also fearful of facing physical as well as verbal violence at the hands of the families of sick patients of COVID-19. They manifested the symptoms of lack of concentration and headache. The personal coping mechanisms that helped them to mollify their distress were the support from their family and strict self-isolation measures. The strategies, which should be adopted according to them, were teaching skills for self-rescue to the front line HCWs, optimal increase in wages, control of misinformation circulation on media, and limits on shift-rotation.

One of the fears faced by the HCWs working directly with COVID-19 patients was violence faced by them at the hands of the patient’s families. It can be due to the rapid deterioration of their health status, the lack of evidence-based guidelines along with continually updated treatment regimens. The violence was partly due to the various misconceptions spread around the general masses regarding this pandemic as well as the limited treatment modalities. Another precipitating factor was the admission of the patients in isolation centers where the families were not allowed to visit them and were informed of the status of their patients by the doctors. The broad range of patient outcomes from mild infection to the requirement of ventilatory support and information spread on a hearsay basis also negatively affected the trust between the care providers and families of the patients. These HCWs also proposed conducting workshops to teach them skills for self-rescue. Skills for self-rescue include learning self emotional dependence. For the healthcare staff working under highly stressful conditions in the pandemic when they are overburdened with drastically increased shift hours and more number of patients to take care of, it is important that they need to be taught skills to rescue themselves from the psychological overburden. It also includes teaching them relaxation exercises in order to cope with their stress and increase their work performance.

Due to the current pandemic and the increased expectations from HCWs, it is no wonder the medical team members are overwhelmed by such prevailing and distressing thoughts that can be the cause of adverse psychological sequelae leading to various somatic symptoms [5]. In accordance with the observation of previous studies that were carried out in different countries (China, Italy, Singapore & India), our study reflected similar types of fears faced by the health care team members based in Pakistan [6-8]. Zerbini et al. reported a similar effect that HCWs in other departments of the hospitals were afraid of getting infected from symptom-free non-tested COVID carriers, the source of infection for others during a symptom-free period [9]. The psychological strain and physical exhaustion contribute to the development of adverse physical as well as psychological outcomes in the form of anxiety, insomnia, and stress headache resulting from working long duty hours at the hospital, lack of concentration, restlessness, fatigue, muscle tension as well as panic attacks. Among the symptoms displayed by the HCWs directly working with COVID-19 patients, they reported headache, which has also been reported by Chew et al., as the most common symptom associated with psychological distress [5]. In addition to headache, lack of concentration experienced by the healthcare workers in both groups was also reported by Song et al. [10]. Moreover, previous studies have significantly reported gender of the health care professionals working in COVID-19 wards as a predictive factor for the development of severe depression, anxiety, and distress, which were more common in female healthcare workers compared to the male healthcare workers. In our study, we found no significant association of depressive symptoms with gender. Possible factors leading to these psychological problems, as reported by Que et al. in their study, are to handle the false information that has been circulating around about the pandemic, deal with the criticism from other frontline workers, being at higher risk of getting infected, and the lack of confidence attributed to the limited knowledge about the disease and its treatment. This indicates the critical role of providing psychological support to health care workers and hence strategies to reduce this psychological burden must be introduced to provide support to the health care workers during this pandemic and even after the pandemic. The personal coping mechanisms that were established in our study, i.e. support from family and colleagues, had also been identified in previous studies. Que et al. from their study results suggested that regular exercise and a higher household income may serve as protective factors against developing depression and similar results have been reported in our study where exercise was used to cope with the stress and increase in the wages of HCWs was proposed as a strategy for administrative authorities [7]. Other strategies that can help in psychological stresses including effective general public education through social, electronic, and print media, as well as reduced working hours, have also been supported by Cai et al. [11]. Blake et al. suggested the use of a digital support and learning package, which includes evidence-based guidance, to check on the psychological well-being of the frontline health care workers and most importantly to support their psychological well-being during and after this pandemic by providing advice from experts in mental health care via direct emails, social media, and professional networks [4].

This study had several limitations. With a limited sample size, it covers only the medical and paramedical staff working in Lahore, Pakistan. Moreover, the study was conducted two months after the pandemic started but some psychological effects take longer to express, and this study could not report on those effects. The representation of the nurses and allied health professionals was also low in our study; further studies should be conducted to answer the questions related to them. A study that can compare the psychological stresses faced by the HCWs with and without the strategies advised can help in ascertaining the efficacy of these suggestions.

## Conclusions

Finally, the findings that were of most significance were the fears of getting themselves infected, putting family members at risk, violence by caretakers of patients, symptoms of tense muscles, lack of concentration, coping mechanisms of proper exercise and strict isolation measures, as well as strategies suggested by HCWs, i.e. teaching skills for self-rescue, optimal increase in wages and information disseminated by media sources and shift-rotation should be considered seriously. All these findings can be implicated in policy-making for future pandemics to reduce the mental stress and anxiety on front-line professionals. However, further studies are needed to elaborate on these effects in other cities of Pakistan.

## Appendices

Study questionnaire

A questionnaire, designed in the form of a Google document, was used as a quantitative data collection tool. It was sent to doctors, nurses, and allied health professionals working at different levels in various hospitals in Lahore, Pakistan. The questionnaire was divided into five sections. The first section consisted of questions related to demographics. The second section contained 10 questions regarding the fears that are being faced by healthcare workers amid the COVID-19 pandemic. The third section was based on the symptoms being experienced by healthcare workers and included eight questions. The fourth section, rooted in different possibilities that help them cope with the stress personally, consisted of 12 questions. The last section focused on strategies that should be adopted by administrative bodies to reduce such psychological issues in the future. This section had 14 questions in total. Each question from all the sections had five choices on a 5-point scale (1 = Strongly agree, 2 = Agree, 3 = Neutral, 4 = Disagree, 5 = Strongly disagree) except the Demographic section and the first two questions of the fourth section, that was answered with only two possibilities (0 = no, 1 = yes).
